# Structural Characterization of TRAF6 N-Terminal for Therapeutic Uses and Computational Studies on New Derivatives

**DOI:** 10.3390/ph16111608

**Published:** 2023-11-14

**Authors:** Omur Guven, Belgin Sever, Faika Başoğlu-Ünal, Abdulilah Ece, Hiroshi Tateishi, Ryoko Koga, Mohamed O. Radwan, Nefise Demir, Mustafa Can, Mutlu Dilsiz Aytemir, Jun-ichiro Inoue, Masami Otsuka, Mikako Fujita, Halilibrahim Ciftci, Hasan DeMirci

**Affiliations:** 1 Department of Molecular Biology and Genetics, Koç University, Istanbul 34450, Turkey; oguven@ku.edu.tr; 2 Medicinal and Biological Chemistry Science Farm Joint Research Laboratory, Faculty of Life Sciences, Kumamoto University, Kumamoto 862-0973, Japan; belginsever@anadolu.edu.tr (B.S.); htateishi@kumamoto-u.ac.jp (H.T.); cherry9pichan@gmail.com (R.K.); motsuka@gpo.kumamoto-u.ac.jp (M.O.); mfujita@kumamoto-u.ac.jp (M.F.); 3 Department of Pharmaceutical Chemistry, Faculty of Pharmacy, Anadolu University, Eskisehir 26470, Turkey; 4 Department of Pharmaceutical Chemistry, Faculty of Pharmacy, European University of Lefke, Northern Cyprus, TR-10, Mersin 99770, Turkey; fabasoglu@eul.edu.tr; 5 Department of Pharmaceutical Chemistry, Faculty of Pharmacy, Biruni University, Istanbul 34015, Turkey; aece@biruni.edu.tr; 6 Department of Nanoscience and Nanotechnology, Izmir Katip Celebi University, Izmir 35620, Turkey; demir.nefise53@gmail.com; 7 Faculty of Engineering and Architecture, Department of Engineering Sciences, Izmir Katip Celebi University, Izmir 35620, Turkey; mustafa.can@ikc.edu.tr; 8 Department of Pharmaceutical Chemistry, Faculty of Pharmacy, İzmir Katip Çelebi University, Izmir 35620, Turkey; mutlud@hacettepe.edu.tr; 9 Department of Pharmaceutical Chemistry, Faculty of Pharmacy, Hacettepe University, Ankara 6100, Turkey; 10 Research Platform Office, The Institute of Medical Science, The University of Tokyo, Tokyo 108-8639, Japan; jun-i@g.ecc.u-tokyo.ac.jp; 11 Department of Drug Discovery, Science Farm Ltd., Kumamoto 862-0976, Japan; 12 Koc University Isbank Center for Infectious Diseases (KUISCID), Koc University, Istanbul 34010, Turkey; 13 Stanford PULSE Institute, SLAC National Laboratory, Menlo Park, CA 94025, USA

**Keywords:** TRAF6, zinc finger, RING domain, cancer, X-ray crystallography, structural biology, Turkish light source “*Turkish DeLight*”, molecular modelling, pharmacokinetic determinants

## Abstract

Tumor necrosis factor receptor-associated factors (TRAFs) are a protein family with a wide variety of roles and binding partners. Among them, TRAF6, a ubiquitin ligase, possesses unique receptor binding specificity and shows diverse functions in immune system regulation, cellular signaling, central nervous system, and tumor formation. TRAF6 consists of an N-terminal Really Interesting New Gene (RING) domain, multiple zinc fingers, and a C-terminal TRAF domain. TRAF6 is an important therapeutic target for various disorders and structural studies of this protein are crucial for the development of next-generation therapeutics. Here, we presented a TRAF6 N-terminal structure determined at the Turkish light source “*Turkish DeLight*” to be 3.2 Å resolution at cryogenic temperature (PDB ID: 8HZ2). This structure offers insight into the domain organization and zinc-binding, which are critical for protein function. Since the RING domain and the zinc fingers are key targets for TRAF6 therapeutics, structural insights are crucial for future research. Separately, we rationally designed numerous new compounds and performed molecular docking studies using this template (PDB ID:8HZ2). According to the results, 10 new compounds formed key interactions with essential residues and zinc ion in the N-terminal region of TRAF6. Molecular dynamic (MD) simulations were performed for 300 ns to evaluate the stability of three docked complexes (compounds 256, 322, and 489). Compounds 256 and 489 was found to possess favorable bindings with TRAF6. These new compounds also showed moderate to good pharmacokinetic profiles, making them potential future drug candidates as TRAF6 inhibitors.

## 1. Introduction

Zinc finger proteins are widely distributed transcription factors in the human genome with an array of biological functions. These functions include those associated with ubiquitin-mediated protein degradation, signal transduction, differentiation, metabolism, apoptosis, autophagy, migration, invasion, and a plethora of other processes. These functions arise from the capability of zinc finger proteins to interact with their particular DNA and RNA targets. Zinc finger proteins are dependent on Zn^2+^ cations, which can bind to cysteine and histidine residues. These proteins can be separated into distinct members of classical and non-classical types, referring to proteins whose zinc fingers contain the signature cys-cys-his-his (C_2_H_2_) motif and those that do not. Despite the great endeavors for the identification of the majority of zinc finger motifs, the structures of most of them have remained poorly characterized [1,2,3,4].

Tumor necrosis factor (TNF) receptor-associated factor (TRAF) family proteins are key regulatory molecules in the immune and inflammatory systems. They are the main signal transducers for the TNF receptor, the Interleukin-1 receptor/Toll-like receptor (IL-1/TLR), and NOD-like receptor (NLR) superfamilies. Until recently, TRAFs were classified as classical members (TRAF1-6) and a single nonclassical member (TRAF7) [5,6,7]. In general, TRAFs (TRAF1-7) adopt a common structure including the Really Interesting New Gene (RING) domain (except for TRAF1), zinc finger motifs, a coiled-coil domain, and a highly conserved C-terminal *β*-sandwich domain (TRAF-C or MATH domain). Apart from TRAF7, other TRAF proteins share a common structure at the TRAF-C domain. The RING domain is responsible for E3 ubiquitin ligase activity structurally supported by the zinc fingers. TRAF6 is a well-characterized E3 ubiquitin ligase, whereas the E3 activity of other TRAFs (TRAF2, TRAF3, and TRAF5) is not fully identified [8,9,10].

TRAF6, a non-conventional E3 ubiquitin ligase, attracts significant attention as the most studied TRAF member, differing from other TRAFs participating in the signal transduction of both the TNF receptor and the IL-1/TLR family proteins. TRAF6 is capable of activating a cascade of signaling events and upstream kinases, including kappa-B kinase (IKK), c-Jun NH_2_-terminal kinase (JNK), and p38 mitogen-activated protein kinase, (MAPK) resulting in the stimulation of transcription factors including interferon-regulatory factor (IRF), the nuclear factor kappa B (NF-κB), and activator protein-1 (AP1) families [11,12,13,14,15]. In particular, NF-κB regulates the expression of a variety of genes involved in inflammatory responses, proliferation, differentiation, migration, cell adhesion, and apoptosis. Thus, NF-κB dysfunction can increase cancer cell proliferation and hamper apoptosis [16,17,18]. Moreover, TRAF6 has been reported to enhance the ubiquitination and activation of protein kinase B (AKT) and transforming growth factor activated kinase 1 (TAK1), leading to cell cycle progression, proliferation, and migration of cancer cells along with impairment of apoptosis in cancer cells. Therefore, the overexpression of TRAF6 is linked with inflammatory disorders and various types of cancers including pancreatic, liver, lung, head and neck, breast, colorectal, prostate, melanoma, and osteosarcoma [19,20,21,22,23,24]. On the other hand, it has also been reported that TRAF6 serves important roles in osteoclastogenesis, defective lymph node organogenesis, and hypohidrotic ectodermal dysplasia [15,25]. Recent studies also documented that high levels of TRAF6 were observed in serum patients with autoimmune diseases including systemic lupus erythematosus, rheumatoid arthritis, and myasthenia gravis [26]. Separately, the connection between upregulation and/or accumulation of TRAF6 and neurodegenerative disorders such as Alzheimer’s disease, Parkinson’s disease, and amyotrophic lateral sclerosis (ALS) has been reported since TRAF6 triggers neuronal apoptosis and central nervous system (CNS) disruption. In CNS, TRAF6 also contributes to inflammatory responses in stroke and neuropathic pain [27,28,29,30].

In this study, we investigated the N-terminal region of TRAF6 at atomic resolution to examine the structure. We designed 503 new compounds on the basis of SN-1, which was previously synthesized by our group and was determined to bind to TRAF6 decreasing long-chain poly-ubiquitination [31,32]. After we determined the structure (PDB ID: 8HZ2), we conducted molecular docking studies for our 503 new SN-1 derivatives using this template (PDB ID: 8HZ2) [33] compared to SN-1 and molecular dynamic (MD) simulations for three favorable docked complexes. We also conducted absorption, distribution, metabolism, and excretion (ADME) analysis to detect the most promising candidates with optimum pharmacokinetic profiles. Our findings have important implications for further pharmaceutical studies, particularly for the development of next-generation TRAF6 inhibitors to be effective as anti-inflammatory, anticancer, anti-osteoporosis, immunosuppressant or anti-neurodegenerative agents.

## 2. Results

### 2.1. TRAF6 N-Terminal Structure at 3.2 Å Resolution

**Figure 1:**
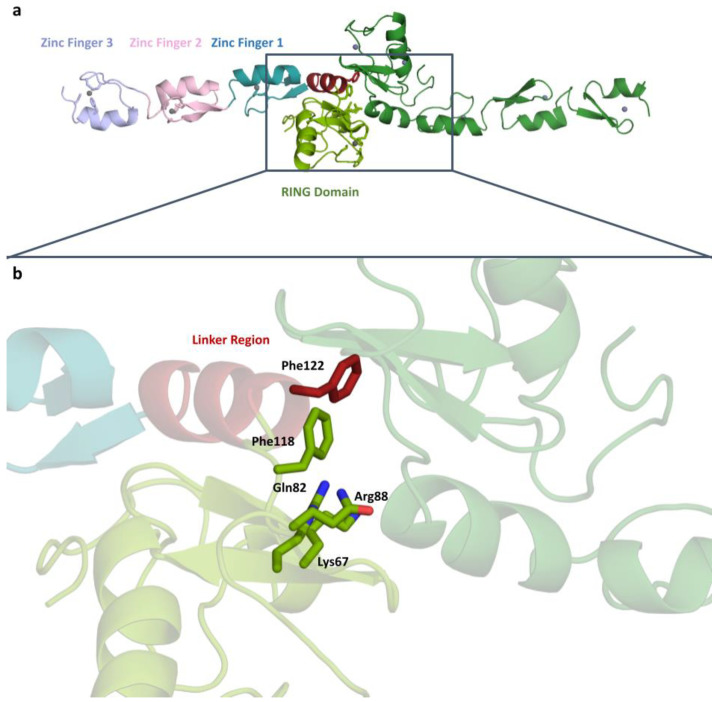
(**a**) Dimeric structure of TRAF6 N-terminal region at 3.2 Å (Chain A RING Domain: Splitpea, ZF1: Deepteal, ZF2: Lightpink, ZF3: Lightblue, Zn: Gray; Chain B: Forest, Zinc atoms: Gray) and domain organization of TRAF6 N-terminal region. (**b**) A close-up of the dimerization residues is shown at the dimerization surface.

TRAF6 is a 59.4 kDa protein consisting of 522 amino acids. Here, we structurally calculated the 18.14 kDa N-terminal region consisting of 157 amino acids. The crystal belongs to the P1 triclinic space group with a = 45.893, b = 51.6293, c = 54.3003, α = 91.064, β = 112.16, γ = 108.43. The dimerized structure of TRAF6 N-terminal region was determined to be 3.2 Å resolution at cryogenic temperature at the Turkish light source “*Turkish DeLight*” [34] (Figure 1). The determined structure was deposited to the PDB database with the ID: 8HZ2 [33].

Our study reveals the structure of TRAF6, which has dimerized at the N-terminal RING domain and linker region. Residues known to mediate the dimerization are shown in Figure 1. There are four residues in the RING domain (Lys67, Gln82, Arg88, and Phe118) and one residue in the linker region (Phe122) shown to be participating in dimerization. 

TRAF6 N-terminal region consists of five domains, a RING domain, a linker helix, and three zinc fingers. Figure 1a shows the domain organization in detail, where the zinc atoms can be clearly seen at the center of the domains.

### 2.2. Detailed Analysis of RING Domain and Zinc Fingers

**Figure 2:**
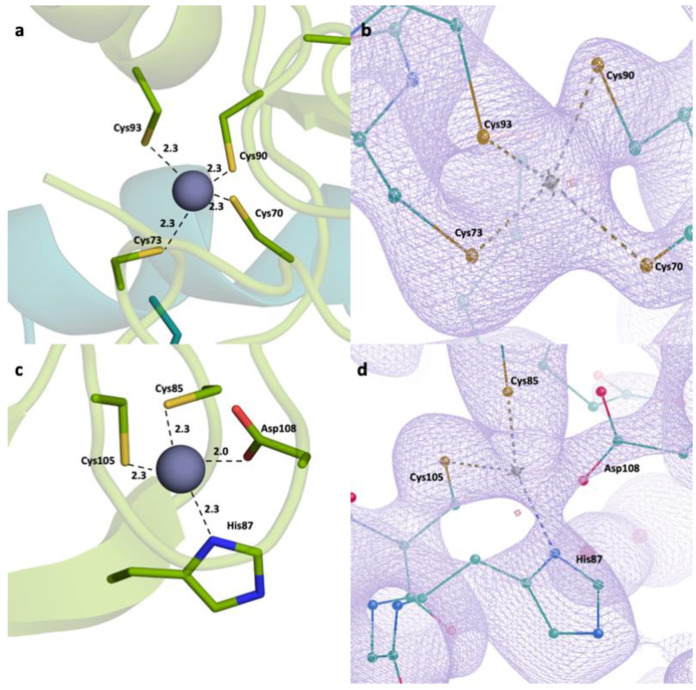
RING domain zinc-interacting residues. (**a**,**c**) Distances between residues and the bivalent zinc ions. (**b**,**d**) The *2Fo-Fc* electron density maps shown at sigma level of 1, with the root mean square deviation (RMSD) showing the interaction.

**Figure 3:**
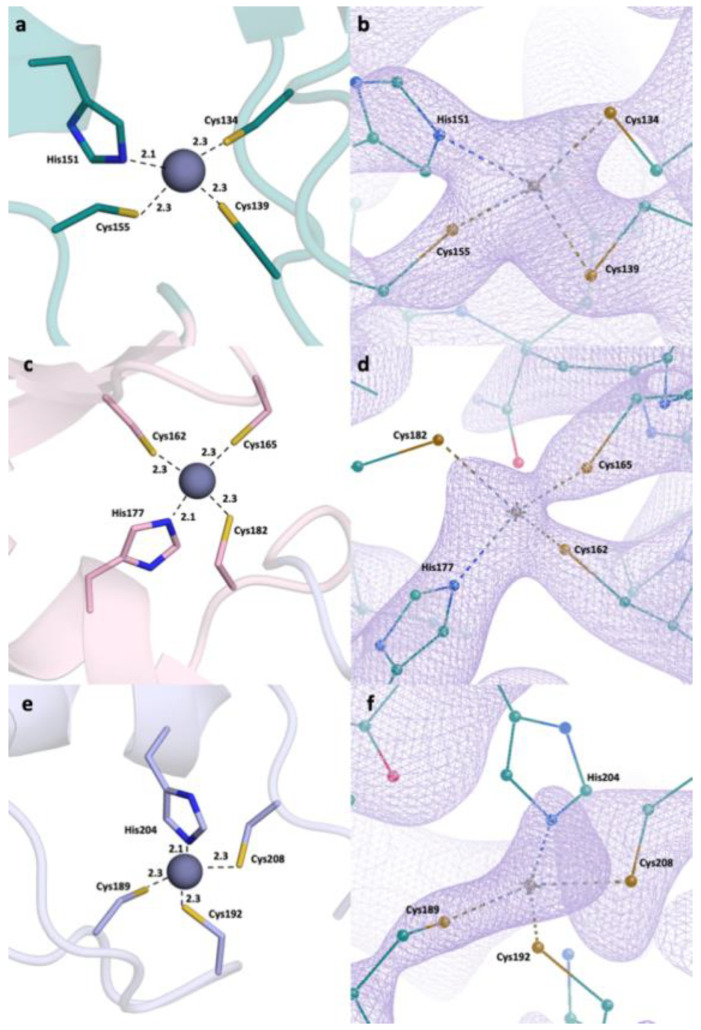
Zinc fingers and zinc-interacting residues. (**a**) Zinc finger 1 distances. (**b**) Zinc finger 1 electron density map at sigma level of 1 RMSD. (**c**) Zinc finger 2 distances. (**d**) Zinc finger 2 *2Fo-Fc* electron density map at sigma level of 1 RMSD. (**e**) Zinc finger 3 distances. (**f**) Zinc finger 3 *2Fo-Fc* electron density map at sigma level of 1 RMSD.

A closer look at the RING domain (Figure 2) and zinc fingers (Figure 3) was also taken. Every zinc atom and their interacting residues are visible within the refined *2Fo-Fc* density map. A clear repeating pattern among the zinc fingers can be observed in secondary structures. Each finger is made of a Sheet-Loop-Sheet-Helix-Loop pattern. Separately, there are always three Cys residues and one His residues forming the finger. While the two cysteine residues are located on the first loop, the third cysteine is located on the second loop and the histidine is located on the helix region. The distances between zinc and interacting residues are also conserved, ranging from 2.0 to 2.3 Å. We have generated the *2Fo-Fc* electron density maps of the zinc and the interacting residues, which show a continuation throughout and enclose the zinc atom and the residues.

### 2.3. Structural Alignment with the Reference Protein

**Figure 4:**
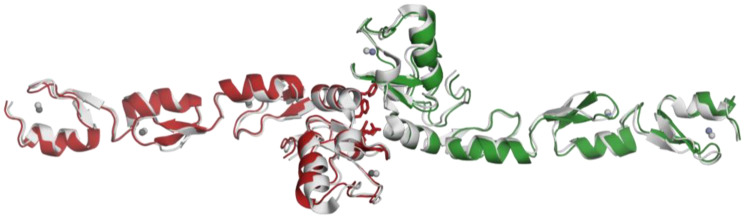
Structure alignment between the obtained data (PDB ID: 8HZ2) and the used model (PDB ID: 3HCS). Model structure is shown in gray.

Our structure (PDB ID:8HZ2) and the reference structure (PDB ID:3HCS) [35] are aligned with the RMSD value of 1.09 and show minor differences within the loops (Figure 4). Also, we have looked at the zinc-interacting residues in both structures (Supplementary Figure S1). Although the residues have similar conformations, there are slight differences based on the loop movements. We have also checked the distances between the zinc-binding residues and zinc ions. Here, we observe that these distances are similar in reference structure and our structure, although the former have a slightly larger range, between 2.1 and 2.8 Å. 

Our structure and the reported structure are almost identical sequence-wise, except for the Histidine tags. The reported structure has a Histidine tag at the C-terminal, while the Histidine tag is cleaved in our structure. Although we did not see a significant difference in the structures, it is an important note for future research.

### 2.4. Design of New Compounds

**Figure 5:**
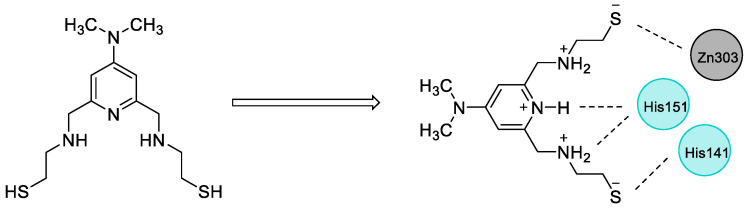
The chemical structure of SN-1 and its favorable interactions with key residues in the N-terminal region of TRAF6.

Over the years, our research group has pursued the discovery of small molecules with efficacy on zinc finger proteins. With great efforts in this area, we have discovered compounds with pyridine and histidine nuclei and proved their remarkable efficacy against zinc finger proteins such as human immunodeficiency virus type I enhancer binding protein 1 (HIV-EP1) [36,37]. Following these efforts, SN-1 (Figure 5), developed by our research group as an inhibitor of zinc finger transcription factor [38], was found to enhance steady-state expression level of antiviral apolipoprotein B mRNA-editing enzyme-catalytic polypeptide-like (APOBEC) 3G (A3G) bearing two zinc-binding domains in the presence of viral infectivity factor (Vif) protein [39]. In our other study, we reported that SN-1 bound to TRAF6, suppressing its auto-ubiquitination and downstream NF-κB signaling. Previous molecular docking study also pointed out that the pyridine ring and NH of the side chain of SN-1 interacted with His151, whereas the dithiol groups interacted with zinc ion and His141 separately in the N-terminal region of TRAF6 (Figure 5) [31]. We further demonstrated that SN-1 derivatives hold promise for developing new drug candidates targeting zinc proteins [32,40,41]. 

**Figure 6:**
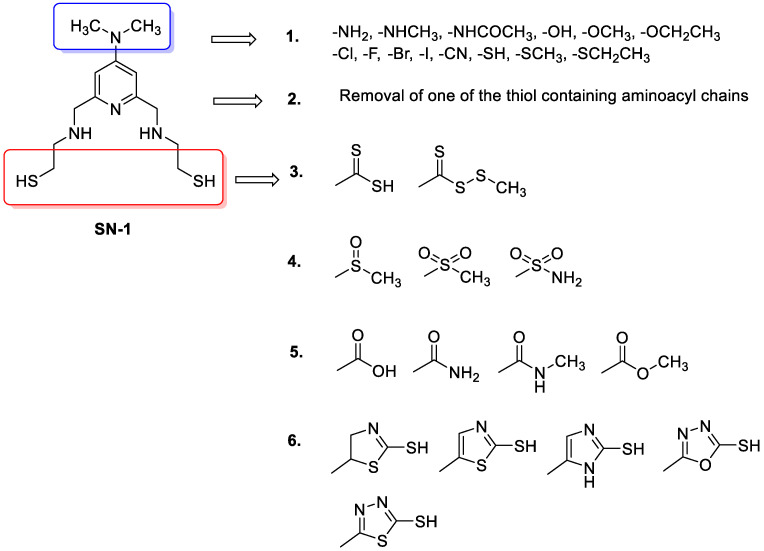
Design strategies for new SN-1 derivatives.

The potential binding mode of SN-1 to the N-terminal region of TRAF6 encouraged us to design 503 new SN-1 derivatives based on six design strategies (Figure 6) on the structural modification of dimethylamino groups on pyridine ring and thiol containing aminoacyl chains.

These strategies are as follows:

- Replacement of dimethylamino groups with amine, methylamine, acetamido, hydroxyl, methoxy, ethoxy, fluoro, chloro, bromo, iodo, cyano, thiol, thiomethyl, thioethyl groups;
- Removal of one of the thiols containing aminoacyl chains;
- Replacement of thiol group to methyl/ethyl (dithioperoxo)thioate, dithiocarbamates in aminoacyl chains;
- Replacement of thiol group to methylsulfinyl, methylsulfonyl, and sulfonamide groups in aminoacyl chains;
- Replacement of thiol group to amide, carboxylic acid, and ester groups in aminoacyl chains;
- Replacement of thiol group to thiol-substituted thiazoline, thiazole, imidazole, oxadiazole, thiadiazole, and pyridine rings in aminoacyl chains.

### 2.5. Molecular Docking Studies for New Compounds

**Figure 7:**
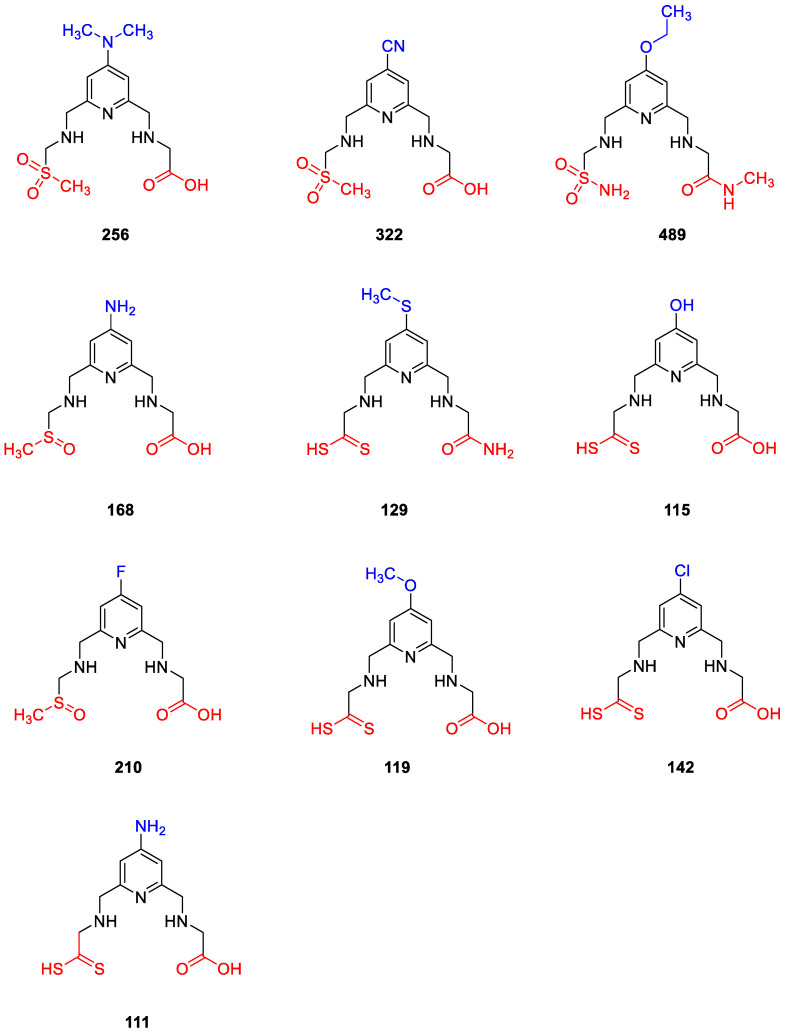
The chemical structures of the most effective new SN-1 derivatives.

**Figure 8:**
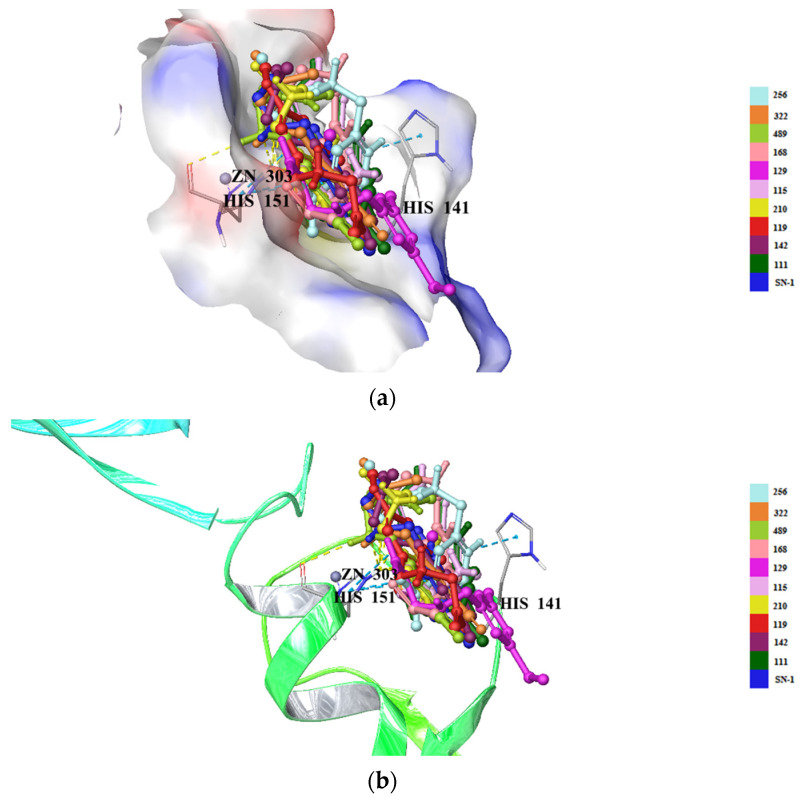
Docking poses of new SN-1 derivatives (yellow dashes: hydrogen bonding; blue dashes: π-π stacking) in the N-terminal region of TRAF6 (PDB ID: 8HZ2) (surface presentation) (**a**) (ribbon presentation) (**b**).

**Figure 9:**
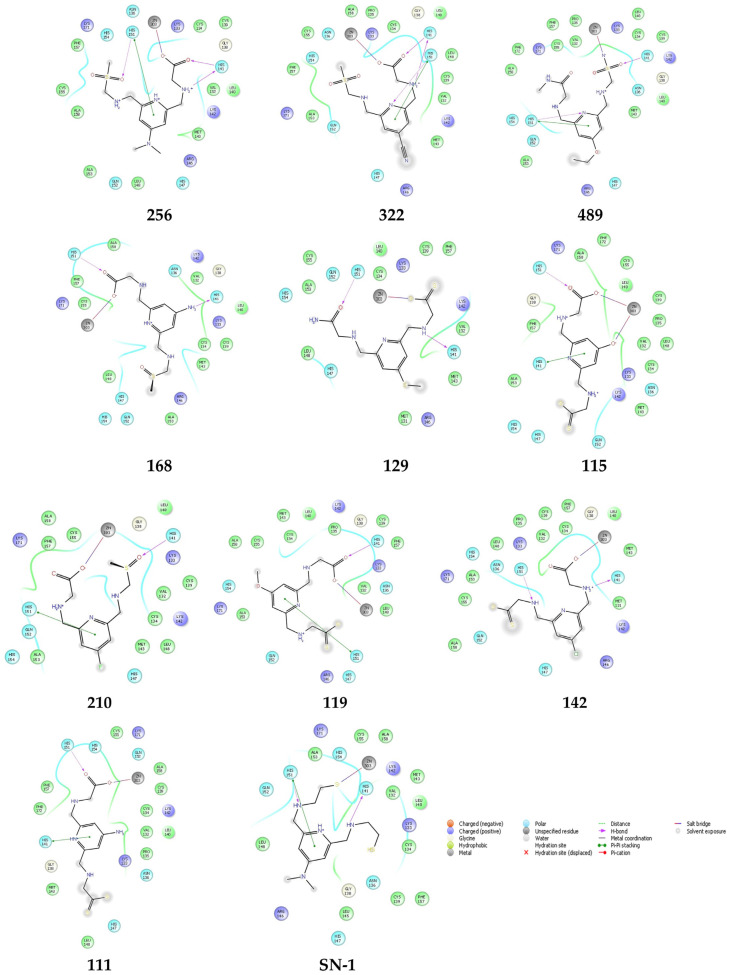
Docking interactions of compounds 256, 322, 489, 168, 129, 115, 210, 119, 142, 111, and SN-1 in the N-terminal region of TRAF6 (PDB ID: 8HZ2).

The crystal structure of the TRAF6 elucidated in the current study was used (PDB IDs: 8HZ2) [33] for 503 new SN-1 derivatives to discover their binding affinities to TRAF6 by molecular docking studies. Results indicated that compounds 111, 115, 119, 129, 142, 168, 210, 256, 322, and 489 (Figure 7) exhibited the highest affinity to the N-terminal region of TRAF6 (Figure 8A,B). These compounds formed key π-π stacking, hydrogen bonding, and ionic bonds with important residues and zinc ion. Although all these compounds revealed a similar binding profile with SN-1 (Figure 8A,B), they showed less affinity compared to SN-1 associated with docking scores (Table 1). Among these derivatives, compounds 256, 322, and 489 were found to bind to TRAF6 more effectively through hydrogen bonding with His141, both π-π stacking, and hydrogen bonding with His151 and salt-bridge formation with zinc ion (Figure 9). Compounds 256, 322, and 489 also presented the highest docking scores as depicted in Table 1. The other compounds were sorted in order of their TRAF6 binding potential as compound 168 > compound 129 > compound 115 > compound 210 > compound 119 > H compound 142 > compound 111.

### 2.6. MD Simulations

MD simulations help us to study dynamics of ligand–protein complexes to elucidate binding interactions and the conformational changes of both protein and the ligands in a biologically simulated environment [42,43,44]. Three complexes (compounds 256, 322, and 489 docked complexes) were subjected to MD simulations to evaluate the stability of macromolecules and also to reveal critical binding interactions throughout the simulations. 

**Figure 10:**
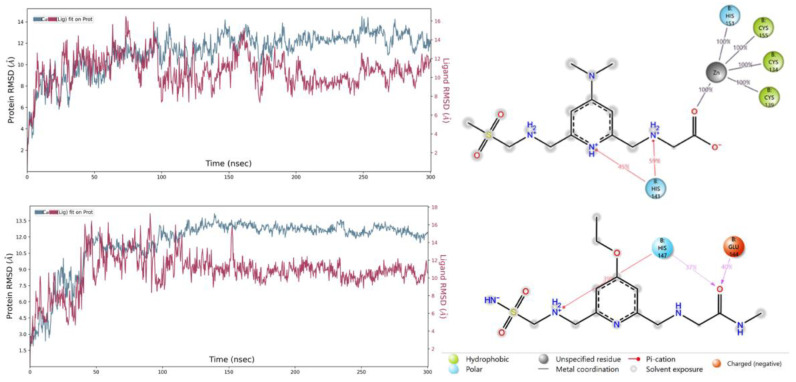
Protein and ligand RMSD (**left** panels) plots and ligand contacts of the compounds 256 (**top**) and 489 (**bottom**) with TRAF6 obtained for a 300 ns simulation.

Figure 10 right panels show ligand–protein contacts for both systems. Only amino acid residues that the ligands interact with for at least 30% of the simulation time are shown. Notably, compound 256 interacts with Zn metal during the whole course of the simulation. A hydrogen bond with His141 is preserved for 59% of the time while another hydrogen bond remains for 45% of all time. Compound 489 acts as a hydrogen bond acceptor and shows favorable interactions with the negatively charged Glu144 and polar His147.

### 2.7. ADME Prediction of New Compounds

Some pharmacokinetic descriptors and properties of these 10 new SN-1 derivatives were predicted using the QikProp algorithm [45] and SwissADME web service [46,47]. These properties involve aqueous solubility (QPlogS), octanol/water partition coefficient (QPlogPo/w), brain/blood partition coefficient (QPlogBB), human serum albumin binding (QPlogKhsa), and compliance to Lipinski’s rule of five in the QikProp algorithm, whereas they involve the potential of several cytochrome P450 (CYP) enzyme inhibitors, P-glycoprotein (P-gp) substrate, BBB permeability, and druglikeness in SwissADME web service. Among all compounds, compound 256 was found to present the most acceptable pharmacokinetic profile. All compounds except for compound 168 exhibited acceptable aqueous solubility with QPlogS values of −1.822 to 0.258 (the limits are −6.5 to 0.5) and human serum albumin binding with QPlogKhsa of −1.194 to −0.244 (the limits are −1.5 to 1.5). The QPlogPo/w values of these derivatives were detected in the specified limits (−2 to 6.5) except for compounds 168, 210, and 322. The QPlogBB values of compounds were found as −2.084 to −0.211 (the limits are −3 to 1.2). However, these negative values and the BOILED-Egg model of SwissADME indicated that these compounds were not capable of crossing the blood brain barrier (BBB). Compounds showed no violation of Lipinski’s rule of five (Table 2). 

**Figure 11:**
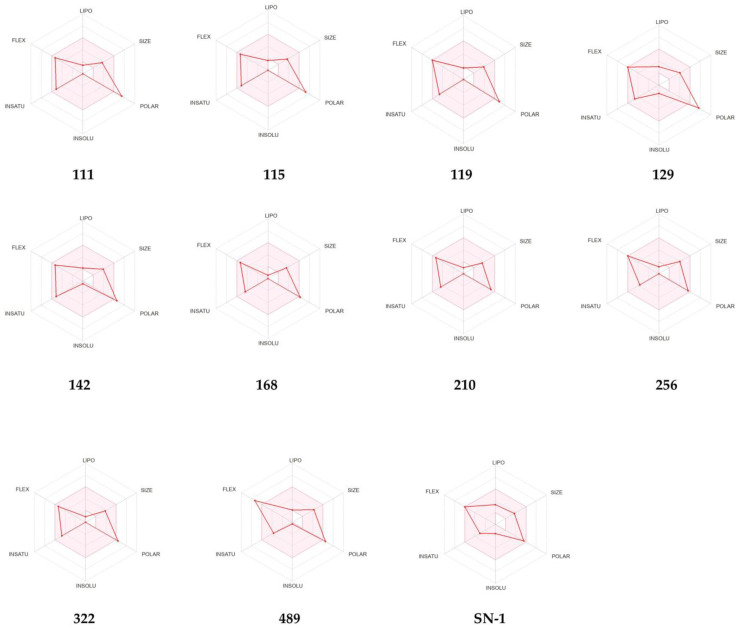
Bioavailability radars for compounds 111, 115, 119, 129, 142, 168, 210, 256, 322, 489, and SN-1 from the SwissADME web tool.

According to SwissADME, the pink region of bioavailability radar (Figure 11) refers to the values of saturation (INSATU), size (SIZE), polarity (POLAR), solubility (INSOLU), lipophilicity (LIPO), and flexibility (FLEX) for oral bioavailability. The red lines for compounds 168, 210, and 256 and SN-1 were detected in the pink area. These compounds also demonstrated good gastrointestinal (GI) absorption. No compounds matched with CYP1A2, CYP2C19, CYP2C9, CYP2D6, and CYP3A4 inhibition. However, compounds 119, 129, 142, and 489 and SN-1 were identified as P-gp substrates.

## 3. Discussion

TRAF6 is widely distributed in the brain, lung, liver, skeletal muscle, and kidney and is involved in a great number of immune and inflammatory reactions as a characteristic E3 ubiquitin ligase. TRAF6 plays a pivotal role in NF-κB stimulation, which triggers a vast array of cellular and organismal processes such as development, immunity, tissue homeostasis and inflammation regulating gene expression, apoptosis, and proliferation at molecular and cellular levels. Therefore, TRAF6 is associated with diverse abnormalities including different cancer types, autoimmune diseases, neurodegenerative disorders, and inflammatory diseases [48,49,50].

Having access to the structural details of TRAF6 provides insights that can support the development of new-generation anticancer therapeutics. Furthermore, the previously deposited structure used as a model (PDB ID: 3HCS) serves as a reference point from which we can observe that our structure is highly similar. The most significant characteristic of TRAF6 is the zinc interaction. Bivalent ions provide a structural scaffold around where the zinc fingers and the RING domain folds. Therefore, zinc has an important role in TRAF6 function, and altering the zinc interaction is a good starting point for inhibition. The overall structure of the TRAF6 N-terminal region shows the dimerized structure with zinc atoms at the center of each domain. The domain organization shows, in addition to RING domain and zinc fingers, a linker region, consisting of a single helix (Figure 3). This region has a role in N-terminal dimerization, as a phenylalanine residue here is part of the dimerization surface. 

RING domain and zinc fingers were analyzed in detail. Zinc fingers are one of the most abundant structural motifs observed in proteins [51]. As their name suggests, they are characterized around a bivalent zinc ion and they can interact with a wide range of molecules, such as nucleic acids and other proteins. Therefore, zinc finger proteins have a wide variety of functions, from transcriptional regulation to actin targeting. Zinc fingers have common patterns: for example, they consist mainly of cysteine and histidine residues, in different ratios [52]. Classical zinc fingers have two of each (Cys_2_His_2_); however, some cases show differences. Zinc finger domains have two β-sheets and one ɑ-helix, although the number of loops change depending on the protein.

RING domain is a common domain in ubiquitin-ligase proteins (E3), with over 340 such proteins possessing this domain. It interacts with two bivalent zinc ions, forming RING fingers [53]. RING domains interact with DNA; therefore, the proteins including RING domain could mediate DNA transcription. The presented structure has a RING domain and three zinc fingers. Each zinc finger follows a conserved pattern: Sheet-Loop-Sheet-Helix-Loop. Moreover, each zinc finger has the same residue pattern: two cysteine residues in the first loop, one histidine residue in the helix, and a third cysteine residue in the second loop. This is an expected result, as the zinc fingers fold around bivalent zinc ions, and they possess similar patterns. This is a classical zinc finger pattern in a way that it has two sheets and a helix; however, it differs as classical zinc finger in its Cys_2_His_2_ structure. 

We have also investigated the distances between the zinc-interacting residues and the zinc ion. We observed that the distances are well within the range of strong bond formation at up to 2.4 Å. The cysteine residues interact with the zinc through the sulfur at the side chain while the histidines interact through their side chain nitrogens. We compared the distances in three zinc fingers, and we observed that they are similar as well, further showing that the zinc fingers have common characteristics. Moreover, it is confirmed that our structure has a RING finger, with its two bivalent zinc ions presenting thus, forming a RING domain. This domain mediates DNA interaction, and is crucial for the role of TRAF6 on NF-κB regulation. These four domains, all revolving around the central zinc atom, are great targets for TRAF6 therapeutics. The high number of cysteine residues results in disulfide bond formation, in addition to zinc interaction; therefore, altering these bonds with reducing agents (for example, dithiol compounds) will go a long way in modulating TRAF6 function.

Structural alignment with the model protein was performed. We used a deposited structure (PDB ID: 3HCS) as a model, and aligned it to the obtained structure (Figure 6). We observed that the structures aligned with high similarity (RMSD = 1.09), except for the loops. This is expected as loops are generally flexible, and the difference is based on the natural properties of the protein. We have also looked into the RING domain and zinc fingers in detail. We observed that the distances between zinc-interacting residues and zinc ions are very similar to that of obtained structure (PDB ID: 8HZ2), ranging between 2.1 and 2.8 Å. This is an expected result as the zinc fingers are conserved regions further confirmed by these distances.

We also applied molecular docking assessment for 503 new SN-1 derivatives. These new compounds were rationally designed for mimicking the binding effects of SN-1, which was previously synthesized and confirmed by our research group to bind to N-terminal region of TRAF6. We also observed that SN-1 and new derivatives presented the similar interactions with our previous study [31] such as hydrogen bonding with His141 and His151 residues and ionic bonding with zinc ion. This similarity confirmed the significance of certain residues in the binding site. Although new compounds revealed similar binding profile with SN-1 (Figure 8A,B), they showed less affinity compared to SN-1 associated with docking scores (Table 1). Among these derivatives, compounds 256, 322, and 489 were found to bind to TRAF6 more effectively through hydrogen bonding with His141, both π-π stacking and hydrogen bonding with His151 and salt-bridge formation with zinc ion These derivatives were designed following different strategies based on the fact that pyridine and amino groups in acyl chains were found critical for binding affinity. The docking assessment on these 503 new derivatives at the N-terminal region of TRAF6 (PDB ID: 8HZ2) supported our previous data. In general, the carboxylate, sulfonamide, and dithiocarbamate groups established salt-bridge formation with zinc ion as similar with the thiol group of SN-1. The replacement of dimethylaminogroup with the other groups did not make a huge impact in binding capacity of new derivatives. The π-π stacking interactions with His151 also played important roles in binding potential of compounds 256, 322 and 489. 

The RMSD plot after MD simulations tells us the average changes in the positions of atoms with respect to a reference frame. At the end of 300 ns MD simulations, compound 322-TRAF6 complex did not stabilize and the ligand was observed to diffuse away from the binding site. The RMSD plots for the 256/TRAF6 and 489/TRAF6 complexes are shown in Figure 10. In both cases, relatively high RMSD values are observed. Compound 256-/TRAF6 complex stabilizes after 200 ns and fluctuations are mostly within 2 Å. Compound 489-/TRAF6 complex, on the other hand, reaches equilibrium after ~105 ns and remains relatively stable until the end of simulation. Visual inspection at the trajectories revealed that the relatively large RMSD values could be explained by fluctuations in the zinc finger regions of the protein that possessed a significant flexibility adopting different conformations. The ring domain and the binding site underwent much fewer fluctuations. We believe that such initial conformational changes were induced by the ligands upon binding. During reorganization and partial folding of the zinc finger regions, both ligands remained in the binding site. 

The ADME calculation results signified that all compounds possessed moderate drug-likeness profiles with appropriate water solubility and lipophilicity values. As the human serum albumin binding is directly related to the volume of distribution and half-life of drugs, it can be concluded that all compounds showed adequate human serum albumin binding values. Although compounds revealed QPlogBB values in the limits, they failed to penetrate into the brain according to BOILED-Egg chart of Swiss ADME. Compounds exerted no inhibition against all tested CYP enzymes indicating a lower risk for drug–drug interactions. However, some compounds were found as P-gp substrates increasing the possibility of resistance by tumor cell lines through efflux. Compound 256 revealed the most prominent ADME values with optimum properties along with high GI absorption implying its drug-likeness potential.

## 4. Materials and Methods

### 4.1. Transformation and Expression

TRAF6 N-Terminal RING domain and 3 zinc fingers with the sequence “MAHHHHHHHHHHVGTENLYFQSMEEIQGYDVEFDPPLESKYECPICLMALREAVQTPCGHRFCKACIIKSIRDAGHKCPVDNEILLENQLFPDNFAKREILSLMVKCPNEGCLHKMELRHLEDHQAHCEFALMDCPQCQRPFQKFHINIHILKDCPRRQVSCDNCAASMAFEDKEIHDQNCPLA” was cloned into pRSF vector with an N-terminal decahistidine purification tag with a TEV cut site. As a cloning restriction enzyme cut sites, *Hin*dIII and *Kpn*I were chosen and the Kanamycin resistance gene was used as a selection marker. The constructed plasmid was transformed into competent *Escherichia coli (E. coli)* BL21 Rosetta-2 strain, with heat shock method. Transformed bacterial cells were grown in 18 L regular LB media containing 50 µg/mL kanamycin and 35 µL/mL chloramphenicol at 37 °C. At OD600 value of 0.8, the protein expression was induced by using β-D-1-thiogalactopyranoside (IPTG) at a final concentration of 0.4 mM for 18 h at 18 °C. Cell harvesting was done using Beckman Allegra 15 R desktop centrifuge at 4 °C at 3500 rpm for 45 min. Cell pellets were stored at −45 °C until protein purification. 

### 4.2. Purification

The cells were dissolved in lysis buffer containing 500 mM NaCl, 50 mM Tris-HCl pH 8, 10% (*v*/*v*) Glycerol, 0.1% (*v*/*v*) Triton X-100, 2 mM BME, and 10 µM ZnCl_2_. The homogenized cells were lysed using a Branson W250 sonifier (Brookfield, CT, USA). The cell lysate was centrifuged at 4 °C at 35000 rpm for 1 h with Beckman Optima^TM^ L-80XP Ultracentrifuge equipped with Ti45 rotor (Beckman, Brea, CA, USA). The pellet containing membranes and cell debris was discarded. The supernatant containing the soluble protein was filtered through 0.2 micron hydrophilic membrane and loaded to a Ni-NTA column that was previously equilibrated with a wash buffer containing 150 mM NaCl, 20 mM Tris-HCl pH 8, 20 mM Imidazole, 10% (*v*/*v*) Glycerol, 2 mM BME, and 10 µM ZnCl_2_. Unbound proteins were discarded by washing the column using a wash buffer. Then, the target protein (TRAF6 RING domain) was eluted using an elution buffer containing 250 mM NaCl, 20 mM Tris-HCl pH 8, 250 mM Imidazole, 10% (*v*/*v*) Glycerol, 2 mM BME and 10 µM ZnCl_2_. Then, the eluted TRAF6 protein was dialyzed in a dialysis membrane (3 kDa MWCO) against a buffer with the same composition as the wash buffer for 3 h at 4 °C to remove excess imidazole. Dialyzed TRAF6 protein was cut using Tobacco Etch Virus nuclear inclusion-a endopeptidase (TEV) protease to remove the hexahistidine-tag overnight at 4 °C.

### 4.3. Crystallization

The crystallization screening of N-terminal decahistidine cleaved TRAF6 was performed using the sitting-drop microbatch under oil method against ~3000 commercially available sparse matrix crystallization screening conditions in a 1:1 volumetric ratio in 72-Terasaki plates (Greiner Bio-One, Kremsmünster, Austria) as described in Ertem 2021 et al. [54]. The mixtures were covered with 16.6 µL 100% paraffin oil (Tekkim Kimya, Istanbul, Türkiye). The crystallization plates were incubated at 4 °C and checked frequently under a stereo light microscope. The best TRAF6 crystals were grown within one month in Salt Rx-I condition #22 (Hampton Research, USA). This condition contains 1.2 M sodium citrate tribasic dihydrate and 0.1 M TRIS-HCl 8.5. 

### 4.4. Crystal Harvesting and Delivery

The TRAF6 crystals were harvested using MiTeGen MicroLoops attached to a magnetic wand [55] while being monitored under microscope [56]. The obtained crystals were flash frozen by plunging in liquid nitrogen and placed in a cryo-cooled sample storage puck (Cat#M-CP-111-021, MiTeGen, USA). Then, the puck was placed into the *Turkish DeLight* liquid nitrogen-filled autosample dewar at 100 K. 

### 4.5. Data Collection and Data Reduction

Collection of diffraction data from the TRAF6 crystal was performed by utilizing Rigaku’s XtaLAB Synergy Flow XRD source “*Turkish DeLight*” at University of Health Sciences (Istanbul, Türkiye) with *CrysAlisPro* software 1.171.42.35a [57]. The crystals were kept cooled by the Cryostream 800 Plus system, which was set to 100 K. The PhotonJet-R X-ray generator operated at 30 mA, 1200.0 W, and 40 kV with 10% beam intensity. The data was collected at 1.54 Å wavelength and the detector distance was set to 47.00 mm. The crystal oscillation width was set to 0.25 degrees per image while exposure time was 20.0 min. CrysAlis Pro version 1.171.42.35a [57] was utilized to perform data reduction and an *.mtz file was obtained as the result.

### 4.6. Structure Determination and Refinement

The cryogenic TRAF6 structure was determined at 3.2 Å with the space group P1 utilizing *PHASER* 2.8.3 [58], an automated molecular replacement program within the *PHENIX* suite 1.20.1 [59]. The previously released TRAF6 structure with PDB ID: 3HCS was used as an initial search model for molecular replacement [35]. Simulated annealing and rigid-body refinements were performed during the first refinement cycle including individual coordinates and translation/liberates/screw (TLS) parameters were refined. The structure was checked by *COOT* [60] after each set of refinement and the solvent molecules were added into unfilled, appropriate electron density maps. The obtained final structure was examined by using *PyMOL* [61] 2.5.4 and *COOT* 0.9.8, and the figures were created. Data collection and refinement statistics were given in Table 3.

### 4.7. Molecular Docking Studies

The crystal structure of the TRAF6 was obtained from the RSCB database (PDB ID: 8HZ2) [33]. The PrepWizard module of Maestro was used for preparing the raw file for the docking analysis. The missing chains were added automatically by Prime and the protonation state was calculated by PropKa at physiological pH. The receptor–ligand complex was minimized by Optimized Potential Liquid Simulations (OPLS_2005) force field. Grid generation of Maestro was used to determine the docking grid. The enclosing box and ligand diameter midpoint box were defined to involve the specified residues (His141, His147, His151, Zn303, Cys134, Cys139, Cys155, Glu156). The generated grid was used for the further docking experiments. Compounds were drawn and cleaned in Maestro workspace and were prepared with energy minimization using OPLS_2005 force field at physiological pH using the LigPrep module. Metal binding states were generated using EpiK. Then, the best minimized structures were submitted to the docking experiments without further modifications. Self-docking experiment was performed to validate the docking protocol. SN-1 was prepared and minimized by the LigPrep module of Maestro using EpiK at physiological pH. The optimum structure (lowest energy) was used for the self-docking procedure. After the obtained ligand was submitted to Glide/SP docking protocols, the same docking procedures were carried out for all designed compounds [62,63].

### 4.8. MD Simulations

The docked complexes of the compounds 256, 322, and 489 were simulated by subjecting to MD simulations using Desmond Software implemented in Schrödinger small molecule drug discovery program [64]. Each complex was put in an orthorhombic box and solvated with the reparameterized transferable intermolecular potential with 4 points model (TIP4P/2005 [65] water model. After neutralizing systems with sodium ions, 0.15 M NaCl was added to meet physiological condition. Those ion and salt additions were excluded within 20 Å of the ligand. Default relaxation protocol and NPT ensemble was selected and finally, 300 ns MD simulation was conducted with the recording interval of 200 ps yielding approximately 1000. 

### 4.9. In Silico ADME Studies

Some crucial pharmacokinetic properties of new SN-1 derivatives were estimated by QikProp module of Maestro [45] and the SwissADME web tool [46,47].

## 5. Conclusions

RING domain and zinc fingers of TRAF6 mediate the activation of nuclear factor kappa B (NF-κB), which has essential roles in the regulation of inflammatory responses, proliferation, differentiation, migration, cell adhesion, and apoptosis. Therefore, it has been found that TRAF6 is overexpressed in various types of cancer including pancreatic, liver, lung, head and neck, breast, colorectal cancers, and melanoma along with inflammatory, autoimmune and neurodegenerative disorders. Therefore, examining and knowing the accurate protein structure can guide us in understanding the exact mechanism of action. The current research manifested the characteristics of the RING domain organization and zinc-binding of TRAF6, shedding light on the crucial functions of the protein. This study encouraged us to carry out further molecular docking studies with TRAF6 and new SN-1 derivatives based on the fact that SN-1 is a potential TRAF6 inhibitor developed by our research group. Results showed that in particular methylsulfonyl and carboxylate carrying compound 256 showed remarkable binding efficacy to the N-terminal region of TRAF6. MD simulations revealed that compound 322 did not form a stable complex while compounds 256 and 489 had favorable bindings with TRAF6. Compound 256 also exhibited appropriate pharmakinetic profile making it as a potential drug-like TRAF6 inhibitor. Overall, the results of this study will lead us for further structural studies including SN-1 and most effective SN-1 derivatives. Based on our continuous endeavors, we aim to develop TRAF6-specific drug candidates to be effective against various disorders. 

## Figures and Tables

**Table 1:** Docking scores (kcal/mol) of the most effective new SN-1 derivatives and SN-1 in the N-terminal region of TRAF6.

Compound	8HZ2
	Docking Score
**256**	−6.889
**322**	−6.747
**489**	−6.259
**168**	−5.698
**129**	−5.647
**115**	−5.578
**210**	−5.570
**119**	−5.532
**142**	−5.526
**111**	−5.522
**SN-1**	−7.335

**Table 2:** Predicted ADME properties of new SN-1 derivatives.

Compound	QPlogS	QPlogPo/w	QPlogBB	QPlogKhsa	Rule of Five
**111**	−1.331	−1.943	−1.128	−0.978	0
**115**	−1.187	−1.972	−0.805	−0.965	0
**119**	−1.822	−0.885	−0.427	−0.798	0
**129**	−0.174	−0.024	−0.462	−0.942	0
**142**	−2.235	−0.552	−0.211	−0.730	0
**168**	0.638	−3.322	−1.680	−1.184	0
**210**	0.258	−2.265	−0.872	−1.059	0
**256**	−1.368	−2.015	−1.648	−0.957	0
**322**	−1.024	−3.418	−1.717	−1.194	0
**489**	0.345	−1.981	−2.084	−1.254	0
**SN-1**	−2.049	2.396	0.624	−0.244	0

**Table 3:** Data collection and refinement statistics.

Dataset	TRAF6
Wavelength (Å)	1.54
Resolution range	20.58–3.231 (3.346–3.231)
Space group	P 1
Unit cell	a = 45.893 Å b = 51.693 Å c = 54.302 Å α = 91.064° β = 112.116° γ = 108.43°
Total reflections	6725 (639)
Unique reflections	4506 (545)
Multiplicity	1.5.(1.5)
Completeness (%)	88.28 (79.80)
Mean I/sigma (I)	7.48 (7.05)
Wilson B-factor	31.96
R-merge	0.6108 (0.6305)
R-meas	0.8208 (0.8537)
R-pim	0.5435 (0.5705)
CC1/2	0.0159 (−0.0765)
CC *	0.177 (−0.407)
Reflections used in refinement	6129 (545)
Reflections used for R-free	606 (56)
R-work	0.2708 (0.3092)
R-free	0.3750 (0.3958)
CC (work)	−0.007 (−0.107)
CC (free)	0.066 (0.380)
Number of non-hydrogen atoms	2537
proteins	2518
ligands	10
solvent	9
Protein residues	314
RMS(bonds)	0.008
RMS(angles)	1.07
Ramachandran favored (%)	90.00
Ramachandran allowed (%)	9.68
Ramachandran outliers (%)	0.32
Rotamer outliers (%)	12.24
Clashscore	19.22
Average B-factor	41.64
macromolecules	41.69
ligands	44.54
solvent	25.02
Number of TLS groups	13

* Statistics for the highest-resolution shell are shown in parentheses.

## Data Availability

The Traf6 N-terminal presented in this manuscript has been deposited to the Protein Data Bank under the accession number 8HZ2 Any remaining information can be obtained from the corresponding author upon request.

## Supplementary Materials

The following supporting information can be downloaded at: https://www.mdpi.com/article/10.3390/ph16111608/s1, Figure S1: A close up of zinc-binding pockets in aligned structure.

## References

[1] Laity J.H., Lee B.M., Wright P.E. (2001). Zinc finger proteins: New insights into structural and functional diversity. Curr. Opin. Struct. Biol..

[2] Jen J., Wang Y.C. (2016). Zinc finger proteins in cancer progression. J. Biomed. Sci..

[3] Gibson T.J., Postma J.P., Brown R.S., Argos P. (1988). A model for the tertiary structure of the 28 residue DNA-binding motif (‘zinc finger’) common to many eukaryotic transcriptional regulatory proteins. Protein Eng..

[4] Bu S., Lv Y., Liu Y., Qiao S., Wang H. (2021). Zinc Finger Proteins in Neuro-Related Diseases Progression. Front. Neurosci..

[5] Zhao L., Hao Y., Song Z., Fan Y., Li S. (2021). TRIM37 negatively regulates inflammatory responses induced by virus infection via controlling TRAF6 ubiquitination. Biochem. Biophys. Res. Commun..

[6] Li J., Liu N., Tang L., Yan B., Chen X., Zhang J., Peng C. (2020). The relationship between TRAF6 and tumors. Cancer Cell Int..

[7] Chen Y., Li Y., Li P.T., Luo Z.H., Zhang Z.P., Wang Y.L., Zou P.F. (2022). Novel Findings in Teleost TRAF4, a Protein Acts as an Enhancer in TRIF and TRAF6 Mediated Antiviral and Inflammatory Signaling. Front. Immunol..

[8] Lamothe B., Campos A.D., Webster W.K., Gopinathan A., Hur L., Darnay B.G. (2008). The RING domain and first zinc finger of TRAF6 coordinate signaling by interleukin-1, lipopolysaccharide, and RANKL. J. Biol. Chem..

[9] He X., Li Y., Li C., Liu L.J., Zhang X.D., Liu Y., Shu H.B. (2013). USP2a negatively regulates IL-1β- and virus-induced NF-κB activation by deubiquitinating TRAF6. J. Mol. Cell Biol..

[10] Lalani A.I., Zhu S., Gokhale S., Jin J., Xie P. (2018). TRAF molecules in inflammation and inflammatory diseases. Curr. Pharmacol. Rep..

[11] Bradley J.R., Pober J.S. (2001). Tumor necrosis factor receptor-associated factors (TRAFs). Oncogene.

[12] Wang P.H., Wan D.H., Gu Z.H., Deng X.X., Weng S.P., Yu X.Q., He J.G. (2011). Litopenaeus vannamei tumor necrosis factor receptor-associated factor 6 (TRAF6) responds to Vibrio alginolyticus and white spot syndrome virus (WSSV) infection and activates antimicrobial peptide genes. Dev. Comp. Immunol..

[13] Walsh M.C., Lee J., Choi Y. (2015). Tumor necrosis factor receptor- associated factor 6 (TRAF6) regulation of development, function, and homeostasis of the immune system. Immunol. Rev..

[14] Inoue J.I., Ishida T., Tsukamoto N., Kobayashi N., Naito A., Azuma S., Yamamoto T. (2000). Tumor necrosis factor receptor-associated factor (TRAF) family: Adapter proteins that mediate cytokine signaling. Exp. Cell Res..

[15] Yamamoto M., Gohda J., Akiyama T., Inoue J.I. (2021). TNF receptor-associated factor 6 (TRAF6) plays crucial roles in multiple biological systems through polyubiquitination-mediated NF-κB activation. Proc. Jpn. Acad. Ser. B Phys. Biol. Sci..

[16] Hayden M.S., Ghosh S. (2004). Signaling to NF-kappaB. Genes Dev..

[17] Park M.H., Hong J.T. (2016). Roles of NF-κB in Cancer and Inflammatory Diseases and Their Therapeutic Approaches. Cells.

[18] Soleimani A., Rahmani F., Ferns G.A., Ryzhikov M., Avan A., Hassanian S.M. (2020). Role of the NF-κB signaling pathway in the pathogenesis of colorectal cancer. Gene.

[19] Middleton A.J., Budhidarmo R., Das A., Zhu J., Foglizzo M., Mace P.D., Day C.L. (2017). The activity of TRAF RING homo- and heterodimers is regulated by zinc finger 1. Nat. Commun..

[20] Qi Y., Pradipta A.R., Li M., Zhao X., Lu L., Fu X., Wei J., Hsung R.P., Tanaka K., Zhou L. (2017). Cinchonine induces apoptosis of HeLa and A549 cells through targeting TRAF6. J. Exp. Clin. Cancer Res..

[21] Khusbu F.Y., Zhou X., Roy M., Chen F.Z., Cao Q., Chen H.C. (2020). Resveratrol induces depletion of TRAF6 and suppresses prostate cancer cell proliferation and migration. Int. J. Biochem. Cell Biol..

[22] Li N., Luo L., Wei J., Liu Y., Haque N., Huang H., Qi Y., Huang Z. (2021). Identification of a new TRAF6 inhibitor for the treatment of hepatocellular carcinoma. Int. J. Biol. Macromol..

[23] Guangwei Z., Zhibin C., Qin W., Chunlin L., Penghang L., Ruofan H., Hui C., Hoffman R.M., Jianxin Y. (2022). TRAF6 regulates the signaling pathway influencing colorectal cancer function through ubiquitination mechanisms. Cancer Sci..

[24] Zhao X., Ren L., Wang X., Han G., Wang S., Yao Q., Qi Y. (2022). Benzoyl-xanthone derivative induces apoptosis in MCF-7 cells by binding TRAF6. Exp. Ther. Med..

[25] Bai S., Zha J., Zhao H., Ross F.P., Teitelbaum S.L. (2008). Tumor necrosis factor receptor-associated factor 6 is an intranuclear transcriptional coactivator in osteoclasts. J. Biol. Chem..

[26] Li T., Li Y., Li J.W., Qin Y.H., Zhai H., Feng B., Li H., Zhang N.N., Yang C.S. (2022). Expression of TRAF6 in peripheral blood B cells of patients with myasthenia gravis. BMC Neurol..

[27] Semmler S., Gagné M., Garg P., Pickles S.R., Baudouin C., Hamon-Keromen E., Destroismaisons L., Khalfallah Y., Chaineau M., Caron E. (2020). TNF receptor-associated factor 6 interacts with ALS-linked misfolded superoxide dismutase 1 and promotes aggregation. J. Biol. Chem..

[28] Huang H., Xia A., Sun L., Lu C., Liu Y., Zhu Z., Wang S., Cai J., Zhou X., Liu S. (2021). Pathogenic Functions of Tumor Necrosis Factor Receptor-Associated Factor 6 Signaling Following Traumatic Brain Injury. Front. Mol. Neurosci..

[29] Lu Y., Cao D.L., Ma L.J., Gao Y.J. (2022). TRAF6 Contributes to CFA-Induced Spinal Microglial Activation and Chronic Inflammatory Pain in Mice. Cell Mol. Neurobiol..

[30] Masperone L., Codrich M., Persichetti F., Gustincich S., Zucchelli S., Legname G. (2022). The E3 Ubiquitin Ligase TRAF6 Interacts with the Cellular Prion Protein and Modulates Its Solubility and Recruitment to Cytoplasmic p62/SQSTM1-Positive Aggresome-Like Structures. Mol. Neurobiol..

[31] Koga R., Radwan M.O., Ejima T., Kanemaru Y., Tateishi H., Ali T.F.S., Ciftci H.I., Shibata Y., Taguchi Y., Inoue J.I. (2017). A Dithiol Compound Binds to the Zinc Finger Protein TRAF6 and Suppresses Its Ubiquitination. ChemMedChem.

[32] Radwan M.O., Koga R., Hida T., Ejima T., Kanemaru Y., Tateishi H., Okamoto Y., Inoue J.I., Fujita M., Otsuka M. (2019). Minimum structural requirements for inhibitors of the zinc finger protein TRAF6. Bioorg. Med. Chem. Lett..

[33] Guven O., Ciftci H., DeMirci H. (2023). Tumor Necrosis Factor Receptor Associated Factor 6 (TRAF6) N-terminal Domain. PDB Entry—8HZ2.

[34] Gul M., Ayan E., Destan E., Johnson J.A., Shafiei A., Kepceoglu A., Yilmaz M., Ertem F.B., Yapici I., Tosun B. (2023). Rapid and efficient ambient temperature X-ray crystal structure determination at Turkish Light Source. Sci. Rep..

[35] Yin Q., Lin S.C., Lamothe B., Lu M., Lo Y.C., Hura G., Zheng L., Rich R.L., Campos A.D., Myszka D.G. (2009). E2 interaction and dimerization in the crystal structure of TRAF6. Nat. Struct. Mol. Biol..

[36] Otsuka M., Fujita M., Aoki T., Ishii S., Sugiura Y., Yamamoto T., Inoue J. (1995). Novel zinc chelators with dual activity in the inhibition of the kappa B site-binding proteins HIV-EP1 and NF-kappa. Br. J. Med. Chem..

[37] Otsuka M., Fujita M., Sugiura Y., Yamamoto T., Inoue J., Maekawa T., Ishii S. (1997). Synthetic inhibitors of regulatory proteins involved in the signaling pathway of the replication of human immunodeficiency virus 1. Bioorg. Med. Chem..

[38] Fujita M., Otsuka M., Sugiura Y. (1996). Metal-chelating inhibitors of a zinc finger protein HIV-EP1. Remarkable potentiation of inhibitory activity by introduction of SH groups. J. Med. Chem..

[39] Ejima T., Hirota M., Mizukami T., Otsuka M., Fujita M. (2011). An anti-HIV-1 compound that increases steady-state expression of apoplipoprotein B mRNA-editing enzyme-catalytic polypeptide-like 3G. Int. J. Mol. Med..

[40] Tanaka A., Radwan M.O., Hamasaki A., Ejima A., Obata E., Koga R., Tateishi H., Okamoto Y., Fujita M., Nakao M. (2017). A novel inhibitor of farnesyltransferase with a zinc site recognition moiety and a farnesyl group. Bioorg. Med. Chem. Lett..

[41] Tateishi H., Tateishi M., Radwan M.O., Masunaga T., Kawatashiro K., Oba Y., Oyama M., Inoue-Kitahashi N., Fujita M., Okamoto Y. (2021). A new inhibitor of ADAM17 composed of a zinc-binding dithiol moiety and a specificity pocket-binding appendage. Chem. Pharm. Bull..

[42] Ece A. (2023). Computer-aided drug design. BMC Chem..

[43] Güleç Ö., Türkeş C., Arslan M., Demir Y., Dincer B., Ece A., Beydemir Ş. (2023). Novel beta-lactam substituted benzenesulfonamides: In vitro enzyme inhibition, cytotoxic activity and in silico interactions. J. Biomol. Struct. Dyn..

[44] Çelik Onar H., Özden E.M., Taslak H.D., Gülçin İ., Ece A., Erçağ E. (2023). Novel coumarin-chalcone derivatives: Synthesis, characterization, antioxidant, cyclic voltammetry, molecular modelling and biological evaluation studies as acetylcholinesterase, α-glycosidase, and carbonic anhydrase inhibitors. Chem. Biol. Interact..

[45] (2016). Schrödinger Release 2016-2: QikProp.

[46] SwissADME. http://www.swissadme.ch.

[47] Daina A., Michielin O., Zoete V. (2017). SwissADME: A free web tool to evaluate pharmacokinetics, drug-likeness and medicinal chemistry friendliness of small molecules. Sci. Rep..

[48] Dou Y., Tian X., Zhang J., Wang Z., Chen G. (2018). Roles of TRAF6 in Central Nervous System. Curr. Neuropharmacol..

[49] Min Y., Kim M.J., Lee S., Chun E., Lee K.Y. (2018). Inhibition of TRAF6 ubiquitin-ligase activity by PRDX1 leads to inhibition of NFKB activation and autophagy activation. Autophagy.

[50] Lin Y., Bai L., Chen W., Xu S. (2010). The NF-kappaB activation pathways, emerging molecular targets for cancer prevention and therapy. Expert Opin. Ther. Targets.

[51] Cassandri M., Smirnov A., Novelli F., Pitolli C., Agostini M., Malewicz M., Melino G., Raschella G. (2017). Zinc-finger proteins in health and disease. Cell Death Discov..

[52] Krishna S.S., Majumdar I., Grishin N.V. (2003). Structural classification of zinc fingers: Survey and summary. Nucleic Acids Res..

[53] Cai C., Tang Y.-D., Zhai J., Zheng C. (2022). The RING finger protein family in health and disease. Signal Transduct. Target. Ther..

[54] Ertem F.B., Guven O., Buyukdag C., Gocenler O., Ayan E., Yuksel B., Gul M., Usta G., Cakılkaya B., Johnson J.A. (2022). Protocol for structure determination of SARS-CoV-2 main protease at near-physiological-temperature by serial femtosecond crystallography. STAR Protoc..

[55] Garman E.F., Owen R.L. (2006). Cryocooling and radiation damage in macromolecular crystallography. Acta Crystallogr. Sect. D Biol. Crystallogr..

[56] Atalay N., Akcan E.K., Gul M., Ayan E., Destan E., Ertem F.B., Tokay N., Çakilkaya B., Nergiz Z., Karakadioğlu G. (2022). Cryogenic X-ray crystallographic studies of biomacromolecules at Turkish Light Source “Turkish DeLight”. Turk. J. Biol..

[57] Rigaku (2021). CrysAlisPro Software System, Version 1.171.42.35a. https://www.rigaku.com.

[58] McCoy A.J., Grosse-Kunstleve R.W., Adams P.D., Winn M.D., Storoni L.C., Read R.J. (2007). Phaser crystallographic software. J. Appl. Crystallogr..

[59] Adams P.D., Afonine P.V., Bunkoczi G., Chen V.B., Davis I.W., Echols N., Headd J.J., Hung L.W., Kapral G.J., Grosse-Kunstleve R.W. (2010). PHENIX: A comprehensive Python-based system for macromolecular structure solution. Acta Crystallogr. D Biol. Crystallogr..

[60] Emsley P., Cowtan K. (2004). Coot: Model-building tools for molecular graphics. Acta Crystallogr. D Biol. Crystallogr..

[61] (2023). The PyMOL Molecular Graphics System, Version 2.5.2.

[62] (2016). Schrödinger Release 2016-2.

[63] Ciftci H., Sever B., Ayan E., Can M., DeMirci H., Otsuka M., TuYuN A.F., Tateishi H., Fujita M. (2022). Identification of New L-Heptanoylphosphatidyl Inositol Pentakisphosphate Derivatives Targeting the Interaction with HIV-1 Gag by Molecular Modelling Studies. Pharmaceuticals.

[64] (2023). Schrödinger Release 2023-3.

[65] Abascal J.L., Vega C. (2005). A general purpose model for the condensed phases of water: TIP4P/2005. J. Chem. Phys..

