# Supplementary figures and images for: Structural Characterization of TRAF6 N-Terminal for Therapeutic Uses and Computational Studies on New Derivatives
Source: Pharmaceuticals (Basel). 2023 Nov 14;16(11):1608. doi: 10.3390/ph16111608 (PMC10674494; doi:10.3390/ph16111608)

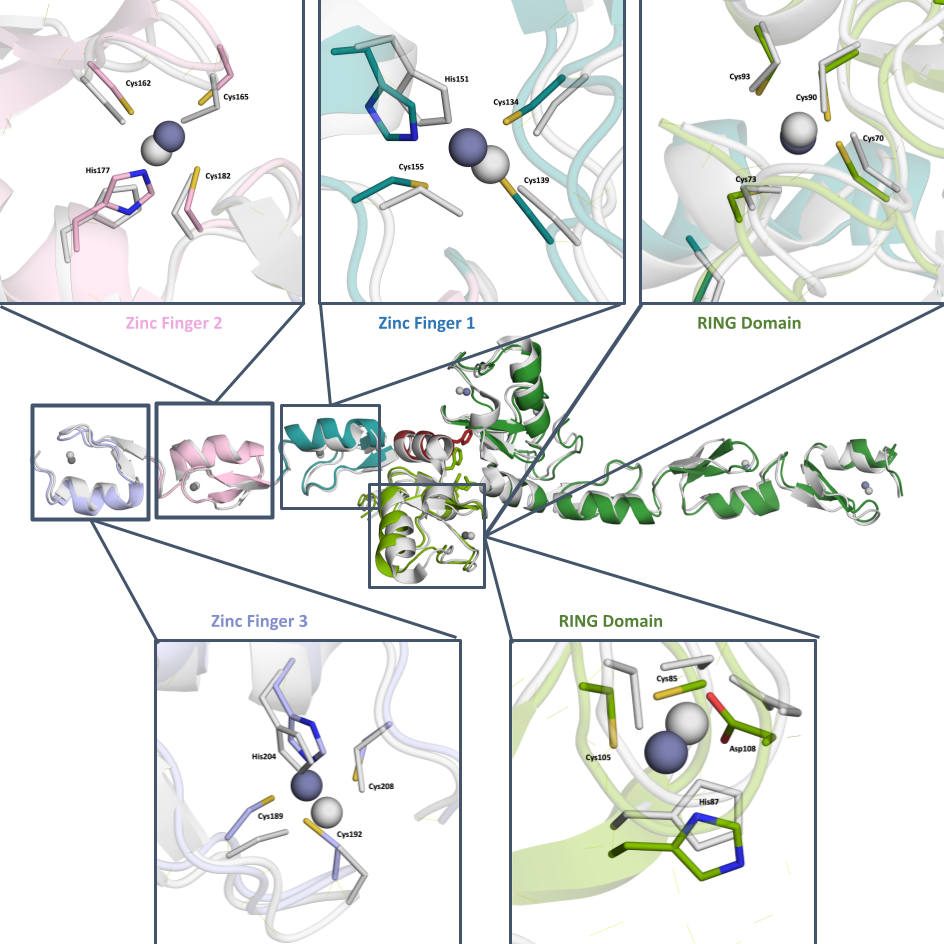

Supplement: Supplementary file 1 [file pharmaceuticals-16-01608-s001.zip › Figure S1.png]
